# Supplementary material for: The interactome of intact mitochondria by cross-linking mass spectrometry provides evidence for coexisting respiratory supercomplexes
Source: Mol Cell Proteomics. 2017 Dec 8;17(2):216–32. doi: 10.1074/mcp.RA117.000470 (PMC5795388; doi:10.1074/mcp.RA117.000470)
Supplement: Supplemental Data [file supp_RA117.000470_133922_0_supp_23978_fzffwf.zip › spectra_annotation/mito_DR_spectra_annotation/352-1-1-1-6-1.pdf]

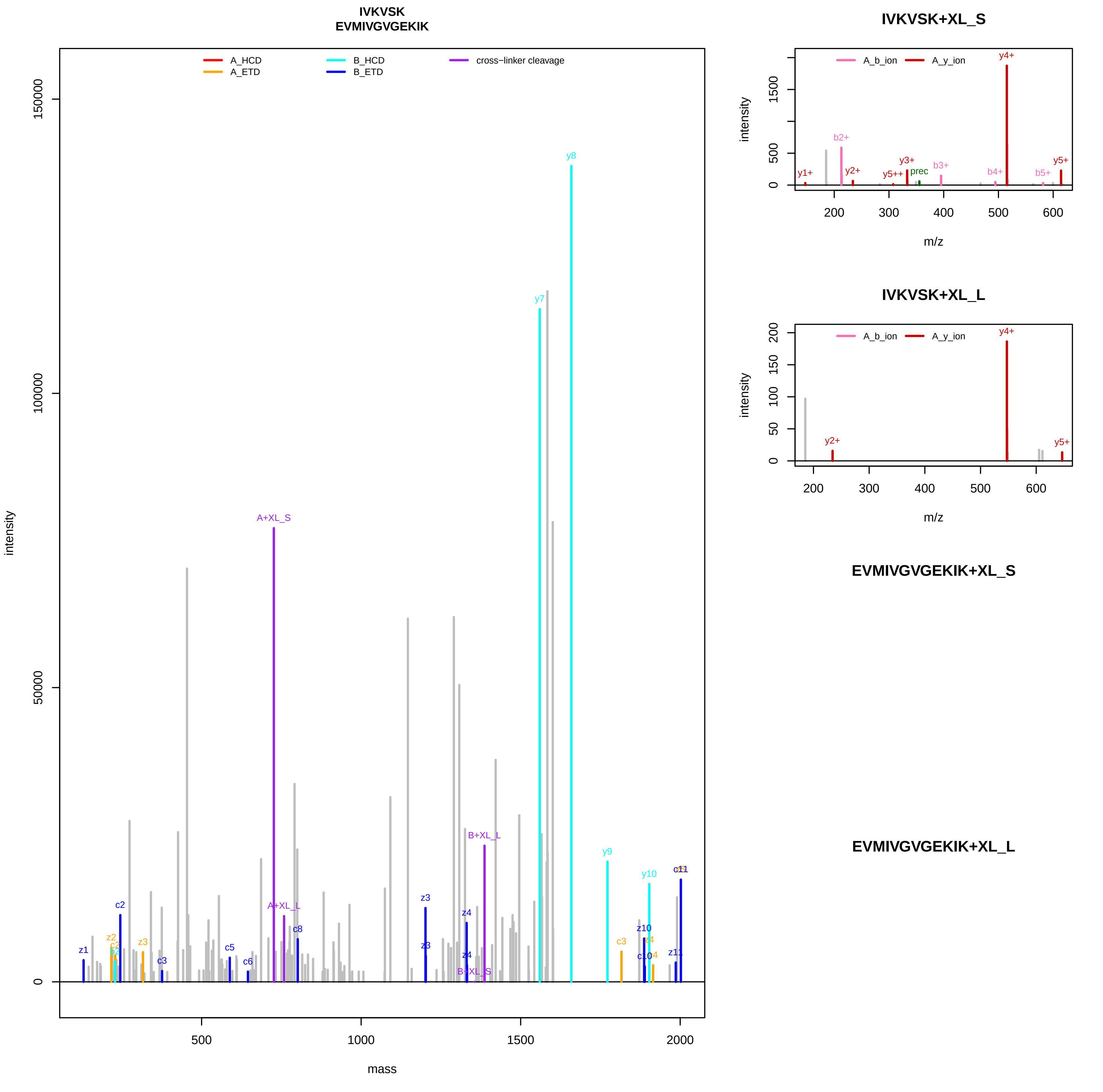
IVKVSK
EVMIVGVGEKIK
A_HCD
A_ETD
B_HCD
B_ETD
cross−linker cleavage
intensity
mass
150000
100000
50000
0
500
1000
1500
2000
y8
y7
A+XL_S
A+XL_L
B+XL_L
B+XL_S
y9
y10
c11
z11
z10
c10
c3
z3
z4
c8
c6
c5
c3
z3
z2
c2
b2
z1
IVKVSK+XL_S
A_b_ion
A_y_ion
y4+
b2+
y2+
y1+
y5++
y3+
prec
b3+
b4+
b5+
y5+
intensity
m/z
IVKVSK+XL_L
A_b_ion
A_y_ion
y4+
y2+
y5+
intensity
m/z
EVMIVGVGEKIK+XL_S
EVMIVGVGEKIK+XL_L
